# Self-reported physical activity and attention performance in children aged 10–11 years

**DOI:** 10.7717/peerj.20867

**Published:** 2026-03-19

**Authors:** Mario Kasovic, Nikola Stračárová, Mateja Očić

**Affiliations:** 1 Department of Physical Activities and Health Sciences, Masaryk University, Faculty of Sport Studies, Brno, Czech Republic; 2 Department of General and Applied Kinesiology, University of Zagreb, Faculty of Kinesiology, Zagreb, Croatia; 3 Laboratory for Sports Games, University of Zagreb, Faculty of Kinesiology, Zagreb, Croatia

**Keywords:** School-aged youth, Exercise and cognition, PAQ-C, d2-R test of attention, Self-reported physical activity, Cognitive functions, Attention

## Abstract

**Background:**

Previous research suggests a possible relationship between physical activity and cognitive functioning in children. However, the findings remain inconsistent, and few studies have examined this link using standardized instruments in preadolescent populations. This study aimed to determine the association between self-reported physical activity and cognitive performance in 10–11-year-old school children.

**Methods:**

A total of 423 children (213 girls and 210 boys; 10.66 ± 0.43 years) participated in this study. The level of physical activity was assessed using the Physical Activity Questionnaire for Children (PAQ-C), while cognitive performance was measured with the d2-R Test of Attention. Data were analyzed using Pearson correlations and a multivariate general linear model (GLM).

**Results:**

Statistically significant correlations were found between PAQ-C scores and two d2-R variables, the total number of items processed (PRZ) and concentration performance (VS), whereas the association with percentage of errors (Ch%) was not significant. The multivariate GLM confirmed these patterns: PAQ-C was significantly associated with PRZ (*β* = 2.596, *p* = 0.003) and VS (*β* = 1.973, *p* = 0.012), but not with Ch% (*p* = 0.281). Gender was also a significant predictor of PRZ and VS.

**Conclusion:**

Self-reported physical activity showed small but statistically significant associations with selected attention outcomes, particularly processing speed and concentration. Given the cross-sectional design and reliance on self-report, causal inference is not possible; however, these findings highlight the potential relevance of physical activity for attentional functioning in school settings and underscore the need for longitudinal and intervention-based research.

## Introduction

Middle childhood (9–12 years) represents a key period of rapid cognitive and attentional development that is closely linked to children’s academic functioning. This age range falls within the broader “Child” category defined by the MeSH database (ages 6–12) and is marked by substantial physiological, cognitive, and psychosocial changes (Newcombe, 2013). This stage is also significant for children’s integration into social structures and increasing academic and behavioral expectations (Eysenck & Keane, 2008).

Physiologically, accelerated growth and hormonal changes may influence emotional expression and interpersonal behavior (Blakemore, Burnett & Dahl, 2010; Kasović et al., 2022). At the neurocognitive level, children show notable advancements in executive functioning—including logical reasoning and abstract thinking—although formal operational thought is still developing (Hillman, Erickson & Kramer, 2008; Piaget & Inhelderová, 2007).

Social and emotional development is equally dynamic, with children showing a more sophisticated understanding of social norms and interpersonal relationships, as well as improved interpretation of emotional cues (Kasović et al., 2022). These developments occur alongside continuous refinement of cognitive processes such as perception, attention, memory, language, problem-solving, and decision-making, which are essential for meeting increasing academic demands (Eysenck & Keane, 2008). These capacities are essential for academic achievement and adjustment to increasingly complex educational demands. In parallel, motor abilities also continue to refine during middle childhood, with improvements in coordination, balance, and overall motor control supporting participation in a range of physical activities (Buttelmann & Karbach, 2017; Kaplan, 2021; Shi & Feng, 2022).

In recent years, scientists have shown growing interest in the link between physical activity and cognitive functioning. Regular physical activity influences not only somatic development but also cognitive performance through several neurobiological mechanisms. For instance, this includes increased cerebral blood flow, enhanced oxygen supply, and modulation of neurotransmitter systems (Seidler et al., 2010; Hillman et al., 2014). Early work (*e.g.*, Young, 1979; Ploughman, 2008) suggested that physical activity may support cognitive functioning through physiological and neuroplastic mechanisms. More recent evidence extends this view, with Mandolesi et al. (2018) highlighting the role of regular activity in promoting neural health and adaptive brain changes. These findings are consistent with recent meta-analyses showing small but reliable benefits of physical activity for executive functions—including inhibitory control, working memory, and selective attention—across school-aged populations (Mao et al., 2024; Vorraber Lawson et al., 2025).

Studies focusing on attention have also shown that regular physical activity improves sustained focus and concentration in school-aged children (Kubesch et al., 2009). Children engaged in structured sports also tend to outperform their sedentary peers in tasks requiring visual selective attention (Alesi et al., 2014). Furthermore, Özüdoğru, Canlı& Yinanç (2022) found that physical fitness has been positively associated with attention levels in young athletes.

Research using brain imaging support these behavioral findings by showing that higher physical fitness is linked to increased hippocampal and basal ganglia volumes—regions involved in memory and executive control (Chaddock-Heyman et al., 2013)—and that appropriately dosed aerobic exercise promotes cognitive resilience (Smith et al., 2010).

Furthermore, motor skills that involve open, strategic, and unpredictable environments, such as team sports, appear to offer greater cognitive benefits than closed skills because they demand rapid adaptation and decision-making (Zhang, 2012; Shi & Feng, 2022). Athletes in open-skill sports also show superior working memory and inhibitory control (Krenn et al., 2018). As noted in the aforementioned review by Vorraber Lawson et al. (2025), cognitively enriched physical activities tend to show more consistent benefits for executive functions than less cognitively demanding aerobic activities. Neuroanatomical adaptations also reflect these differences. Wu et al. (2013) observed that basketball players displayed greater gray matter volume in regions linked to visual-motor decision-making, while badminton players showed changes in fine motor control areas. Higher physical fitness has additionally been linked to greater white matter integrity in prefrontal and temporal regions crucial for attention regulation (Esteban-Cornejo et al., 2019). Importantly, increased participation in physical activity does not compromise academic achievement and may instead support broader cognitive development (Trudeau & Shephard, 2008).

As screen time increases in modern childhood routines, physical inactivity poses risks to somatic, mental, and cognitive functioning (Davranche et al., 2018). Recent large-scale reviews consistently show that, although associations between physical activity and cognitive outcomes in children are modest, they are reliable—particularly for attention and executive-function measures (Mao et al., 2024; Vorraber Lawson et al., 2025). Additional evidence indicates that habitual physical activity is linked to executive functioning and academic achievement (Esteban-Cornejo et al., 2019). Moderate activity levels may also support attentional efficiency (Van der Fels et al., 2015), while the overall effects of physical activity on cognitive outcomes remain small but consistent across studies (Vasilopoulos et al., 2023). Together, these findings highlight the relevance of examining this relationship during preadolescence using accessible, school-feasible assessment tools. Self-report instruments such as the Physical Activity Questionnaire for Children (PAQ-C), are particularly valuable in large school-based samples where objective monitoring is often impractical, yet capturing habitual activity patterns remains essential for understanding variability in cognitive performance. Large population-based studies have further emphasized the value of standardized assessment frameworks for physical activity–related outcomes in children and adolescents, providing normative benchmarks for interpreting developmental variability (Štefan et al., 2022).

The present study is grounded in this growing body of evidence and focuses specifically on selective attention, a core component of cognitive functioning with particular relevance for school performance. Therefore, the aim of this study was to examine the relationship between self-reported physical activity and selective attention performance in children aged 10 to 11 years.

## Materials & Methods

### Participants

This cross-sectional study included a total of 423 fifth-grade students (213 girls and 210 boys), aged 10 to 11 years (10.66 ± 0.43 years), from 12 elementary schools in the city of Brno, South Moravian Region, Czech Republic. The selection of participants was conducted using a convenience sampling method from schools that voluntarily agreed to participate. The final sample size was determined pragmatically based on the number of schools that chose to participate in the project. A sensitivity analysis in G*Power showed that, with *n* = 423, *α* = .05 and 80% power, the study was adequately powered to detect small correlations between physical activity and attention. Body height and body mass were measured using a standard portable stadiometer and a calibrated digital scale, and BMI was calculated accordingly. These anthropometric variables were collected solely for descriptive purposes to characterize the sample.

### Measures

#### Physical activity

The Physical Activity Questionnaire for Children (PAQ-C) was used to assess participants’ physical activity levels. This self-administered, 7-day recall questionnaire includes nine items related to participation in different physical activities during the school week. Each item is scored on a 5-point Likert scale, and the final PAQ-C score is calculated as the mean of all items, with higher scores indicating greater physical activity. The PAQ-C does not differentiate between intensity levels but provides a general indicator of habitual physical activity over the past week. The questionnaire has been previously validated for use in children aged 8 to 14 and demonstrates acceptable reliability and construct validity (Kowalski et al., 2004). A validated Czech version of the PAQ-C is also available and has shown good psychometric properties in Czech schoolchildren (Cuberek, Janíková & Dygrýn, 2021).

#### Attention performance

Attention and concentration were assessed using the d2-R Test of Attention. This paper-and-pencil test measures selective attention and mental concentration under time pressure. Participants are required to scan rows of characters and cross out all instances of the target stimulus (“d” with two marks) while ignoring distractors. Key outcome variables include:

•  Total number of processed items (PRZ),

•  Concentration performance (VS), and

•  Percentage of errors (Ch%).

The d2-R test is a standardized and validated instrument, commonly used to assess attention and concentration in children and adolescents (Brickenkamp, Kirchner & Leger, 2010).

### Procedure

The assessment was conducted during regular school hours in a quiet classroom environment. Each participant completed both the PAQ-C and the d2-R test individually, under the supervision of trained research assistants. The PAQ-C required approximately 10 min to complete, whereas the d2-R test lasted about 8 min. Researchers assisted the participants with the PAQ-C questionnaire by helping them interpret the questions where necessary. The same test order was used for all participants: PAQ-C first, followed by the d2-R test. All assessments were conducted under standardized conditions using uniform instructions and consistent testing procedures. Classroom teachers were present to supervise but did not assist with the responses. Only children with parental consent forms participated in the study.

### Statistical analysis

All statistical analyses were performed using IBM SPSS Statistics version 29.0. Descriptive statistics (mean, standard deviation, minimum and maximum) were calculated for all variables. Normality of distributions was evaluated using skewness and kurtosis indices. Values within ±1 were considered acceptable and indicative of approximate normality. Pearson correlation coefficients were computed to determine the strength and direction of the association between physical activity (PAQ-C score) and the d2-R variables (PRZ, VS and Ch%). To further examine the relationship between physical activity and attention performance, a multivariate general linear model (GLM) was conducted with the three d2-R outcomes (Ch%, PRZ, VS) entered simultaneously as dependent variables. PAQ-C score and gender were included as predictors. This approach allowed us to account for shared variance between the attention measures and to adjust for potential gender-related differences in attention performance. Univariate parameter estimates (B coefficients, standard errors, t values, and *p* values) were used to evaluate the unique associations of PAQ-C and gender with each attention outcome. Statistical significance was set at *p* < 0.05. All participants completed both instruments in full; no missing data were recorded.

### Ethics statement

The study was conducted in accordance with the ethical standards of the institutional research committee and the Declaration of Helsinki. Participation was voluntary and anonymous. Written informed consent was obtained from the parents or legal guardians of all participants. Ethical approval was granted by the Ethics Committee of the Masaryk University (Approval No: EKV-2018-042).

## Results

Table 1 presents participant basic characteristics by sex. The total sample consisted of 423 children (213 girls and 210 boys) aged 10 to 11 years. The overall mean age of the participants was 10.66 ±  0.43 years. Mean body mass was 39.86 ± 9.28 kg, with BMI values ranging from 13.10 to 29.60 kg/m^2^. Girls and boys showed very similar averages for body mass and BMI.

**Table 1:** Participant characteristics. Descriptive statistics about the participants (age, body mass, BMI).

Participants	N	Age (years)	Body mass (kg)			BMI (kg/m^2^)		
		M ± SD	M ± SD	MIN	MAX	M ± SD	MIN	MAX
Girls	213	10.56 ± 0.35	40.00 ± 7.40	24.20	73.70	18.23 ± 3.2	13.10	28.20
Boys	210	10.78 ± 0.48	39.68 ± 9.40	24.30	70.30	18.25 ± 3.03	13.10	29.60
Total sample	423	10.66 ± 0.43	39.86 ± 9.28	24.20	73.70	18.24 ± 3.16	13.10	29.60

Table 2 shows skewness and kurtosis values for the variables included in the main analysis. All variables show values close to zero, suggesting a distribution close to normal and justifying the use of parametric statistical procedures in subsequent analyses. Descriptive results for the attention test included a mean error percentage (Ch%) of 103.06 ± 11.88, processed relevant characters (PRZ) 104.88 ±  11.64, and concentration performance (VS) 103.39 ± 10.65. The average PAQ-C score was 2.96 ± 0.71, indicating a moderate level of physical activity.

**Table 2:** Descriptive statistics for the Physical Activity Questionnaire for Children (PAQ-C) and d2-R test of attention.

Test	N	M ± SD	MIN	MAX	SKEW	KURT
PAQ-C	423	2.96 ± 0.71	0.87	4.76	0.08	−0.10
Ch%	423	103.80 ± 12.02	70	130	0.10	0.01
PRZ	423	104.88 ± 11.60	70	130	−0.10	0.12
VS	423	103.39 ± 10.65	70	130	−0.19	0.28

**Notes.**

PAQ-C Physical Activity Questionnaire for Children Ch% error percentage PRZ number of processed relevant items VS concentration performance

Table 3 presents the correlation matrix between physical activity levels, measured using the PAQ-C, and three variables from the d2-R attention test: error percentage (Ch%), number of processed relevant items (PRZ), and concentration performance (VS). Pearson’s correlation coefficients showed a statistically significant but weak positive correlation between the PAQ-C score and several attention test variables. Specifically, the PAQ-C score was positively associated with the number of processed relevant items (PRZ; *r* = 0.155, *p* = 0.005), indicating a weak but statistically significant correlation (*p* < 0.01), as well as with concentration performance (VS; *r* = 0.130, *p* = 0.018), also showing a weak but significant positive correlation (*p* < 0.05). No statistically significant correlation was found between PAQ-C and Ch% (*r* = 0.058, *p* = 0.294), suggesting that physical activity levels were not associated with the error rate. These findings indicate that children with higher self-reported physical activity levels tended to process more relevant items and achieve slightly higher concentration performance scores, although the associations were weak.

**Table 3:** Correlation matrix between physical activity level and attention performance variables.

Variable	Ch%	PRZ	VS
PAQ-C	0.0577	0.1549	0.1302
	*p* = 0.294	*p* = 0.005^*^	*p* = 0.018^*^

**Notes.**

PAQ-C Physical Activity Questionnaire for Children Ch% error percentage PRZ number of processed relevant items VS concentration performance

* *p* < 0.05.

A multivariate GLM was conducted to examine whether self-reported physical activity (PAQ-C) and gender were associated with attention performance outcomes from the d2-R test (Table 4). At the univariate level, PAQ-C was not significantly associated with error percentage (Ch%; *p* = 0.281), indicating no meaningful relationship between physical activity and error rate. In contrast, PAQ-C showed statistically significant positive associations with both the number of processed relevant items (PRZ; *p* = 0.003) and concentration performance (VS; *p* = 0.012). Gender was also a significant predictor of PRZ (*p* = 0.008) and VS (*p* = 0.002). Given that gender was coded as 1 = boys and 2 = girls, these positive coefficients indicate that girls scored approximately 2–3 points higher than boys on both processing speed and concentration measures. No significant gender effect was observed for Ch% (*p* = 0.318). Overall, the GLM results suggest that, after accounting for gender differences, higher self-reported physical activity is associated with slightly better performance on processing speed and concentration (PRZ and VS), whereas no association was observed for error rate (Ch%).

**Table 4:** Multivariate general linear model for attention outcomes (Ch%, PRZ, VS) with PAQ-C and gender as predictors.

Dependent variable	Parameter	*β*	Std. error	t	(*p*)
Ch%	PAQ-C	1.014	0.939	1.080	0.281
	gender	0.984	0.984	1.000	0.318
PRZ	PAQ-C	2.596	0.879	2.953	0.003^*^
	gender	2.448	0.921	2.658	0.008^*^
VS	PAQ-C	1.973	0.785	2.512	0.012^*^
	gender	2.583	0.823	3.138	0.002^*^

**Notes.**

Gender was coded as 1 = boys and 2 = girls.

PAQ-C Physical Activity Questionnaire for Children Ch% error percentage PRZ number of processed relevant items VS concentration performance

* *p* < 0.05.

## Discussion

The primary aim of this study was to examine the relationship between physical activity levels and selected aspects of cognitive function—specifically attention and concentration—in children aged 10 to 11 years. The results showed that higher levels of self-reported physical activity were associated with better performance in two attention-related tasks, the number of correctly processed symbols (PRZ) and concentration performance (VS), as measured by the d2-R test. In contrast, no association was observed between physical activity and error percentage (Ch%). These findings were consistent across both the correlational analyses and the multivariate GLM.

While these results are encouraging, the associations were modest in magnitude, indicating that physical activity explains only a small proportion of variance in attentional performance. The GLM further revealed that gender was a significant predictor of PRZ and VS, with girls scoring approximately 2–3 points higher than boys. This finding highlights the importance of accounting for demographic variables when examining attentional outcomes. It is also important to note that this study’s cross-sectional design does not permit causal inference and cannot be directly compared with intervention studies, which typically report stronger effects. The present findings therefore reflect correlational patterns observed within a naturalistic school-based context.

Several neurophysiological mechanisms proposed in prior literature may underlie the observed associations. Regular physical activity has been shown to increase cerebral blood flow and oxygen delivery to brain regions responsible for attention regulation, while also promoting neuroplastic adaptations that support more efficient cognitive processing. Additionally, physical activity may help modulate arousal and stress levels through improved regulation of catecholamines and cortisol, which can facilitate sustained attentional performance in school-aged children (Lubans et al., 2016).

The idea that physical activity may be associated with cognitive functioning in children is supported by numerous studies suggesting that physically active children tend to demonstrate not only improved physical fitness but also enhanced psychological well-being and cognitive abilities. Given the complexity of children’s growth and development, there is a continuous need for empirical research exploring how increased levels of physical activity influence cognitive performance, particularly in domains such as attention and executive function.

In this study, attention was assessed using standardized d2-R measures (PRZ, VS, Ch%), while physical activity was evaluated using the PAQ-C questionnaire. These indicators were then used to examine the associations between habitual physical activity and attentional performance. Correlation analysis revealed statistically significant and positive associations between physical activity levels and both the number of correctly processed symbols (PRZ) (*r* = 0.15, *p* = 0.005) and concentration performance (VS) (*r* = 0.13, *p* = 0.018). However, the correlation between physical activity and error percentage (Ch%) was weak and not statistically significant (*r* = 0.058, *p* = 0.294). These correlational findings aligned with the GLM results, which additionally adjusted for gender. Overall, the pattern suggests a trend toward a positive relationship, though the strength of the associations remained small, consistent with prior research on habitual physical activity and attention. Future studies may consider modified testing procedures or larger, stratified samples to further investigate this relationship.

The multivariate GLM, which simultaneously modelled all three attention outcomes with PAQ-C and gender as predictors, confirmed that physical activity was significantly associated with PRZ (*β* = 2.596, *p* = 0.003) and VS (*β* = 1.973, *p* = 0.012), but not with Ch% (*β* = 1.014, *p* = 0.281). Gender also emerged as a significant predictor of PRZ (*β* = 2.448, *p* = 0.008) and VS (*β* = 2.583, *p* = 0.002), indicating meaningful differences between boys and girls on these attention-related measures. This pattern aligns with previous work indicating that attentional processes such as processing speed and task efficiency are interrelated cognitive components in childhood (Abrahamse et al., 2015), which may help explain their shared variance with physical activity indicators.

Children who regularly participate in sport or structured physical activities often show better performance on tests involving working memory, an essential function for efficient information processing and academic success. One of the proposed mechanisms behind this effect is improved cerebral blood flow and increased oxygen and glucose supply to brain regions during and after physical activity. Meta-analyses also point to enhanced inhibitory control as a result of regular physical exercise—a cognitive function strongly linked to self-regulation and executive processing (Bidzan-Bluma & Lipowska, 2018).

The results of this study are consistent with those of Trudeau & Shephard (2008), who reported that an increased number of physical education sessions per week improved student performance and classroom behavior without negatively impacting academic outcomes in other areas. Similarly, Janssen & LeBlanc (2010) found that physical activity positively affects psychological health by reducing depressive symptoms and anxiety while improving self-confidence—factors which indirectly support cognitive development.

The present findings also reinforce conclusions from Davis et al. (2011) and Kamijo et al. (2011), who demonstrated that physical activity interventions contribute to the development of executive functions in children, particularly in the areas of planning, attention, and working memory. Donnelly et al. (2016) also observed that physical activity in children aged 5 to 13 years is associated with improved cognitive performance. Because the current study did not employ an intervention design, these comparisons should be interpreted cautiously; however, they provide useful context for understanding the cognitive domains in which associations with physical activity often emerge.

However, in our study, the association between PAQ-C and VS (concentration performance) was weaker compared to PRZ. This may be partially attributed to individual variability in concentration performance across participants. Nonetheless, the observed correlation still indicates a positive trend, which has been previously confirmed in systematic reviews, especially those examining the effects of acute bouts of physical activity on cognitive outcomes.

Prior research has also shown that different aspects of physical activity (type, frequency, intensity) may have distinct effects on various dimensions of cognitive performance. For example, studies conducted by Scudder et al. (2014) and Donnelly et al. (2016) reported that children with higher aerobic fitness demonstrated superior inhibitory control and greater task accuracy, both of which are essential for effective learning and academic achievement.

Although intervention studies such as FITKids (Hillman et al., 2014) demonstrate the potential cognitive benefits of structured physical activity programs, these findings cannot be directly compared with the correlational results observed in the present study. Rather, they highlight that physical activity may support aspects of executive functioning, including attention and cognitive flexibility, under controlled intervention conditions. This interpretation aligns with evidence from meta-analyses showing that structured physical activity programs can produce small-to-moderate improvements in executive functions (Álvarez-Bueno et al., 2017; Vazou et al., 2019). Additional analyses further indicate that the magnitude of cognitive benefits depends strongly on activity type, with cognitively enriched or combined programs yielding the most consistent effects on working memory, inhibition, and cognitive flexibility (Song et al., 2022).

Taken together, the current results align with a growing body of evidence showing associations between physical activity and cognitive outcomes in children. The studies reviewed in this discussion consistently show benefits in executive functions following physical activity interventions, particularly in domains such as attention, working memory, and cognitive flexibility. Our findings add to this literature by demonstrating small but statistically significant associations between self-reported physical activity (PAQ-C) and objective measures of attentional processing and concentration (PRZ and VS) obtained through the d2-R test. These findings support the idea that accessible physical activity screening tools and cognitive assessments can together inform strategies for supporting cognitive development in educational contexts. In practical terms, the associations observed in this study suggest that physical activity and attentional readiness may be linked in everyday classroom settings, indicating that opportunities for movement—such as short active breaks or unstructured outdoor play—could hold potential benefits for children’s engagement and focus. A strength of this study is the large, age-homogeneous sample and the use of validated and widely applied instruments (PAQ-C and d2-R), which increases the reliability and ecological validity of the observed associations.

### Limitations

Several limitations of this study should be acknowledged. First, the use of convenience sampling from schools in a single region may limit the generalizability of the findings. Because participation relied on schools that voluntarily agreed to take part, some degree of selection bias cannot be ruled out. In addition, the use of self-reported data to assess physical activity levels (PAQ-C) introduces the possibility of recall bias and social desirability bias, particularly in younger populations. Although gender was included as a covariate in the GLM analysis, several other potentially important confounding variables—such as socioeconomic status, sleep patterns, nutrition, academic achievement and body mass index—were not assessed and may have influenced the observed associations. Second, the cross-sectional design prevents causal inference; although significant associations were observed, it is not possible to determine the directionality of the relationship between physical activity and cognitive performance. Third, while the d2-R test is a validated instrument for assessing attention, it captures a limited scope of cognitive domains and does not account for broader executive functions such as planning, inhibition, or working memory. Additionally, the relatively weak correlation observed for some measures (*e.g.*, Ch%) suggests that other moderating or mediating variables may play a role and were not controlled in this study. Future research employing longitudinal or intervention-based designs with more comprehensive cognitive assessments and objective measures of physical activity is recommended to better understand the underlying mechanisms.

## Conclusions

The results of this study provide additional evidence of modest associations between self-reported physical activity and specific aspects of attentional performance in school-aged children. Specifically, higher levels of physical activity were associated with better processing speed and concentration performance, although the magnitude of these effects was small. These findings are consistent with prior research suggesting that physical activity and cognitive functioning may be related, particularly in domains involving attention and executive processes. In practical terms, the associations observed in this study suggest that opportunities for regular physical activity—such as short active breaks, regular physical education lessons, or unstructured outdoor play—may be relevant for supporting attentional readiness in classroom settings and children’s overall daily functioning.

Findings from this study highlight the potential relevance of integrating structured physical activity into the daily routines of school-aged children, suggesting that targeted movement programs may represent one component of broader strategies to support attention and cognitive functioning in educational settings. This underscores the potential value of promoting physical activity not only for physical health, but also for optimizing cognitive development during critical developmental periods.

Future research should explore these associations using longitudinal and intervention-based designs, and incorporate additional potential moderating variables such as socioeconomic status, sleep patterns, or specific characteristics of physical activity.

##  Supplemental Information

10.7717/peerj.20867/supp-1 Supplemental Information 1 Raw data Raw scores from the Physical Activity Questionnaire for Children (PAQ-C) and the d2-R Test of Attention, collected from all study participants. This includes a metadata sheet that provides definitions of all variables, including measurement procedures, coding, units, and sample sizes.

10.7717/peerj.20867/supp-2 Supplemental Information 2 STROBE checklist

## Additional Information and Declarations

### Data Availability

The following information was supplied regarding data availability: The raw data are available in the Supplemental Files.
