# Supplementary material for: Self-reported physical activity and attention performance in children aged 10–11 years
Source: PeerJ. 2026 Mar 19;14:e20867. doi: 10.7717/peerj.20867 (PMC13006003; doi:10.7717/peerj.20867)
Supplement: Supplemental Information 2 [file peerj-14-20867-s002.docx]

| **Item** | **Recommendation** | **Reported in manuscript (section)** |
| --- | --- | --- |
| **Title and abstract** |  |  |
| 1 | Indicate the study design with a commonly used term in the title or the abstract | Title; Abstract |
| 2 | Provide an informative and balanced summary of what was done and what was found | Abstract |
| **Introduction** |  |  |
| 3 | Explain the scientific background and rationale for the investigation | Introduction |
| 4 | State specific objectives, including any prespecified hypotheses | Introduction, end |
| **Methods** |  |  |
| 5 | Describe the study design and the rationale | Methods, Study design |
| 6 | Give the setting, locations, and relevant dates, including periods of recruitment, exposure, follow-up, and data collection | Methods, Participants |
| 7 | Give the eligibility criteria, and the sources and methods of selection of participants | Methods, Participants |
| 8 | Clearly define all outcomes, exposures, predictors, potential confounders, and effect modifiers. Give diagnostic criteria if applicable | Methods, Measures (PAQ-C; d2-R Test) |
| 9 | For each variable of interest, give sources of data and details of methods of assessment (measurement) | Methods, Measures |
| 10 | Describe any efforts to address potential sources of bias | Methods, Procedures; Ethical considerations |
| 11 | Explain how the study size was arrived at | Methods, Participants |
| 12a | Describe all statistical methods, including those used to control for confounding | Methods, Statistical analysis |
| 12b | Describe any methods used to examine subgroups and interactions | Methods, Statistical analysis |
| 12c | Explain how missing data were addressed | Methods, Statistical analysis (“no missing data”) |
| 12d | If applicable, describe analytical methods taking account of sampling strategy | Not applicable |
| 12e | Describe any sensitivity analyses | Not applicable |
| **Results** |  |  |
| 13a | Report numbers of individuals at each stage of study (e.g. potentially eligible, examined for eligibility, included in the study, completing follow-up, and analysed) | Results, Participants |
| 13b | Give reasons for non-participation at each stage | Not applicable (all participants included) |
| 13c | Consider use of a flow diagram | Not applicable |
| 14a | Give characteristics of study participants (e.g. demographic, clinical, social) and information on exposures and potential confounders | Results, Table 1 |
| 14b | Indicate the number of participants with missing data for each variable of interest | Results, noted “no missing data” |
| 14c | Summarize follow-up time (e.g. average and total amount) | Not applicable (cross-sectional) |
| 15 | Report numbers of outcome events or summary measures | Results, Tables 2–4 |
| 16a | Give unadjusted estimates and, if applicable, confounder-adjusted estimates and their precision (e.g. 95% CI). Make clear which confounders were adjusted for and why they were included | Results, Tables 2–4 |
| 16b | Report category boundaries when continuous variables were categorized | Not applicable |
| 16c | If relevant, consider translating estimates of relative risk into absolute risk | Not applicable |
| 17 | Report other analyses done—e.g. analyses of subgroups and interactions, sensitivity analyses | Results, Regression analysis |
| **Discussion** |  |  |
| 18 | Summarize key results with reference to study objectives | Discussion, first paragraph |
| 19 | Discuss limitations of the study, taking into account sources of potential bias or imprecision | Discussion, Limitations |
| 20 | Give a cautious overall interpretation of results considering objectives, limitations, multiplicity of analyses, results from similar studies, and other relevant evidence | Discussion, final section |
| 21 | Discuss the generalizability (external validity) of the study results | Discussion, Implications |
| **Other information** |  |  |
| 22 | Give the source of funding and the role of the funders | Funding statement |
